# Cx32 inhibits the autophagic effect of Nur77 in SH-SY5Y cells and rat brain with ischemic stroke

**DOI:** 10.18632/aging.203526

**Published:** 2021-09-22

**Authors:** Fengfeng Ping, Chao Zhang, Xue Wang, Yan Wang, Danli Zhou, Jing Hu, Yanhua Chen, Jingjing Ling, Jia Zhou

**Affiliations:** 1 State Key Laboratory of Natural Medicines, China Pharmaceutical University, Nanjing 211198, China; 2 Department of Good Clinical Practice, The Affiliated Wuxi Children's Hospital of Nanjing Medical University, Wuxi 214023, China; 3 Department of Reproductive Medicine, Wuxi People's Hospital Affiliated to Nanjing Medical University, Wuxi 214023, China; 4 Wuxi School of Medicine, Jiangnan University, Wuxi 214122, China

**Keywords:** Cx32, autophagy, Nur77, mitophagy, ischemic stroke

## Abstract

The pathogenesis of cerebral ischemia-reperfusion (I/R) is complex. Cx32 expression has been reported to be up-regulated in ischemic lesions of aged human brain. Nevertheless, the function of Cx32 during cerebral I/R is poorly understood. Autophagy is of vital importance in the pathogenesis of cerebral I/R. In the current study, we found that oxygen-glucose deprivation/reoxygenation (OGD/R) or I/R insult significantly induced the up-regulation of Cx32 and activation of autophagy. Inhibition of Cx32 alleviated OGD/R or I/R injury, and further activated autophagy. In addition, Nur77 expression was found to be up-regulated after OGD/R or I/R. After inhibiting Cx32, the expression of Nur77 was further increased and Nur77 was translocated from nucleus to mitochondrial. Inhibition of Cx32 also activated mitophagy by promoting autophagosome formation and up-regulating the expression of mitochondrial autophagy marker molecules. Of note, in the siNur77-transfected cells, the number of dysfunctional mitochondrial was increased, and mitophagy was suppressed, which aggravated OGD/R-induced neuronal injury. In conclusion, Cx32 might act as a regulatory factor of Nur77 controlling neuronal autophagy in the brains. Understanding the mechanism of this regulatory pathway will provide new insight into the role Cx32 and Nur77 in cerebral ischemia, offering new opportunities for therapeutics.

## INTRODUCTION

Connexins (Cx) belong to a family of structurally related transmembrane proteins that express in all human organs and tissues. They assemble to form gap junctions in vertebrates [1–2]. To date, 21 connexins have been discovered [3–4], of which 11 connexins are found in the central nervous system [5]. Despite of certain cell specificity, Connexin32 (Cx32) has been found to be abundantly expressed in mammalian brains [6]. When liver or brain tissue is damaged, the expression level of Cx32 is increased. After application of Cx32 inhibitor, these damages could be alleviated by mitigating oxidative stress and apoptosis [7–8]. Oguro et al. reported the up-regulation of Cx32 and Cx36 protein expression in hippocampus after global ischemia. Cx32 knock-out mice was more vulnerability during global ischemia [9]. These findings above suggest that Cx32 may play a crucial role in the cell survival after ischemia. However, to the best of our knowledge, the mechanism of Cx32 underlying its protective role in cerebral ischemia has not been elucidated.

Cerebral ischemia/reperfusion (I/R) injury usually occurs after cardiac arrest, shock and reperfusion of acute cerebral infarction, causing serious damage to brain function [10–11]. Autophagy has been demonstrated to be involved in the occurrence and development of cerebral ischemia injury [12–13]. Although autophagy plays a key role in maintaining cytoplasmic homeostasis, its biological effects have two sides [14]. Under physiological conditions, moderate autophagy is induced after ischemic brain injury. It balances cell division and cell degradation, and promotes the regulation of cell composition recycling. However, excessive autophagy can destroy this balance and induce non-apoptotic programmed death, which is commonly known as II type of programmed cell death [15]. Thus, no consensus is reached on the role of autophagy in cerebral I/R injury, which needs further investigation. In keratocystic odontogenic tumors, a certain correlation has been demonstrated between the change of Cx32 protein level and autophagy [16]. Therefore, we hypothesized that Cx32 participates in cerebral I/R through the regulation of autophagy. Nur77 is a member of the orphan nuclear receptor family. It is widely distributed in the central nervous system, including cortex, hippocampus, striatum, SNpc and other brain regions [17]. Nur77 participates in the pathophysiological process of many diseases, including schizophrenia and Parkinson’s disease [18]. Nevertheless, the function of Nur77 in cerebral ischemia has rarely been investigated. It is reported that Nur77 translocation from nucleus to mitochondrial may result in autophagy or apoptosis [19]. Therefore, it is hypothesized that Nur77 might be involved in Cx32-induced autophagy after cerebral I/R.

The purpose of this study was to investigate the role of Nur77 in Cx32-induced autophagy following OGD/R or I/R insult. This regulatory mechanism may be of great value in the brain after ischemic injury.

## RESULTS

### OGD/R induced up-regulation of Cx32 expression and activation of autophagy

**Figure 1:**
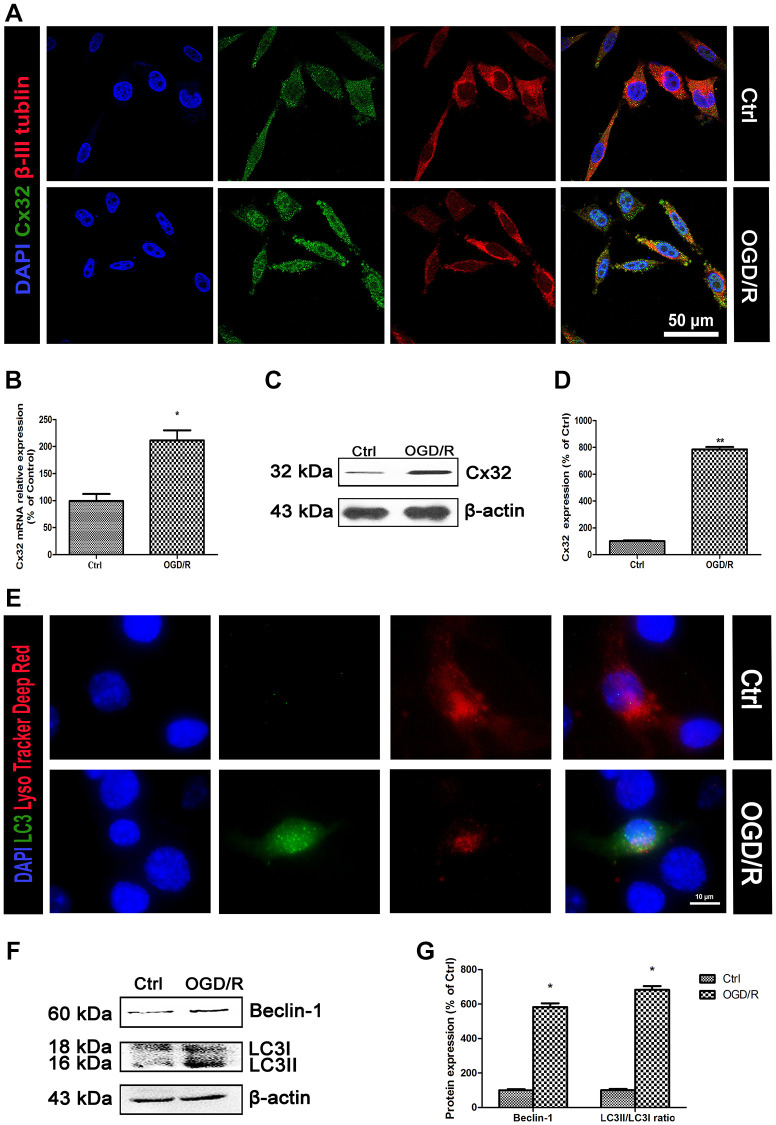
**OGD/R induced up-regulation of Cx32 expression and activation of autophagy.** (**A**) Double labeling of Cx32 and β-tubulin in the Ctrl or OGD/R (2h OGD following 24 h recovery) group. Scale bars, 50 μm. (**B**) The mRNA level of Cx32 was detected by qPCR. (**C**–**D**) Representative bands of Cx32 protein in the cells after OGD/R. (**E**) The autophagosome was labeled with LC3 (green), autophagolysosome was marked by LysoTracker probes (red), and cell nuclei were counterstained by DAPI (blue). Scale bars, 20 μm. (**F**–**G**) Representative bands of Beclin1 and LC3 protein in SH-SY5Y cells after OGD/R. Variation in protein loading was determined by blotting with an anti-β-actin antibody. Densitometric scanning of band intensities were calculated as means ± SD (*n* = 3). ^*^p < 0.05 vs. Ctrl group, ^**^p < 0.01 vs. Ctrl group.

To investigate whether Cx32 can be expressed in the SH-SY5Y cells, double immunostaining of Cx32 and β-III Tubulin-a neuron marker was performed. As shown in Figure 1A, Cx32 was expressed in the normal SH-SY5Y cells, whereas β-III Tubulin was detected in the same time. Compared to the Ctrl group, the expression of Cx32 in the OGD/R group was significantly elevated. PCR and Western blot analysis confirmed this result (Figure 1B–1D).

As shown in Figure 1E, LC3-an autophagic hallmark protein expression was obviously increased in the OGD/R group. Western blot analysis also found that the up-regulation of Beclin-1 and LC3II expression from SH-SY5Y cells appeared after OGD/R injury (Figure 1F–1G). These data indicated that OGD/R induced up-regulation of Cx32 expression and activation of autophagy.

### Inhibition of Cx32 activated autophagy after OGD/R injury

**Figure 2:**
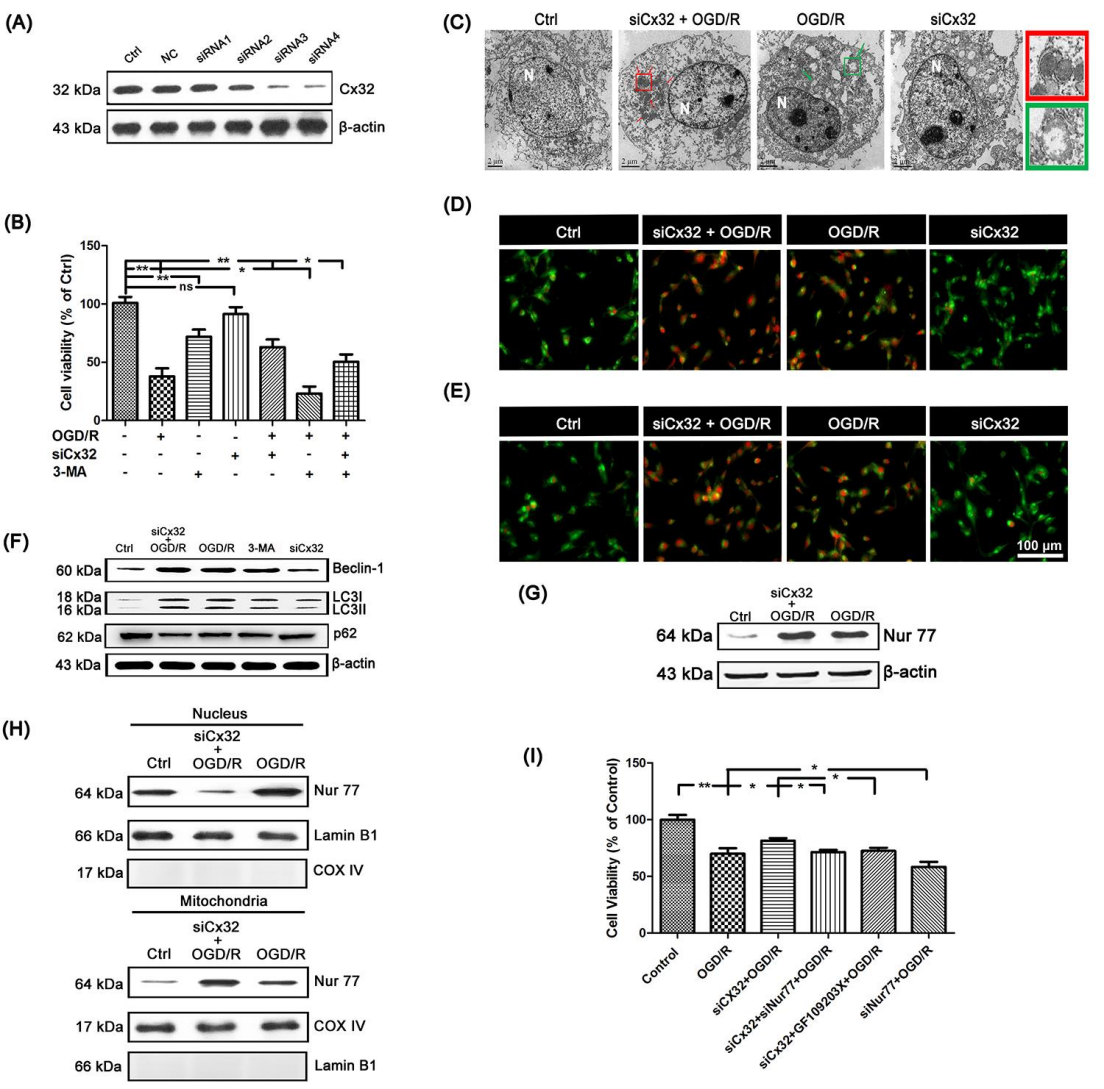
**Inhibition of Cx32 activated autophagy and promoted Nur77 translocating from nucleus to mitochondria after OGD/R injury.** (**A**) After transfection with control siRNA or Cx32 siRNA for 24 h, the cells were collected for Western blots of Cx32. Cells were transfected with Cx32 siRNA for 24 h followed by OGD/R (2 h OGD following by 24 h recovery) before harvested. (**B**) A MTT assay was used for cell viability. (**C**) Images acquired by transmission electron microscope. The arrow marks autophagic vacuoles. Scale bars, 2 μm. (**D**–**E**) Images of Monodansylcadaverine (MDC) staining and acridine orange (AO) staining under fluorescence microscope. Scale bars, 100 μm. (**F**) Representative bands of Beclin1, LC3 and p62 protein in siCx32 cells after OGD/R. (**G**) Representative bands of Nur77 protein in the cells after OGD/R. Variation in protein loading was determined by blotting with an anti-β-actin antibody (*n* = 3). (**H**) Mitochondrial translocation of Nur77 was examined after OGD/R. Mitochondria Nur77 expression was normalized against COX IV. Nucleus Nur77 expression was normalized against α-tubulin expression. (**I**) A MTT assay was applied for the cell viability. Data are expressed as means ± SD (*n* = 6). ^**^p < 0.01, ^*^p < 0.05.

To explore the role of Cx32 after OGD/R injury, SH-SY5Y cells was transfected with Cx32-specific siRNA. Cx32-siRNA4 was chosen for further experiment since it is the most effective at blocking out Cx32 expression (Figure 2A). MTT assay was applied to investigate the cell viability after OGD/R. As shown in Figure 2B, there was a significant decrease of viability for OGD/R-treated cells. The cytotoxic effect was alleviated in siCx32 cells after OGD/R injury, indicating that siCx32 played a protective role in OGD/R.

To elucidate the relationship between Cx32 and autophagy after OGD/R injury, transmission electron microscopy was used to detect autophagy. As shown in Figure 2C, compared to the Ctrl group, more autophagic vacuoles was found in the cytoplasm of cells in the OGD/R group, which was further increased after transfection with Cx32siRNA. In addition, after OGD/R injury, far more red spots and stronger punctate fluorescence of MDC appeared in the cytoplasm of cells, which was strengthened in siCx32 cells (Figure 2D). OGD/R-induced increment of AVO was also reinforced in siCx32 cells (Figure 2E). Furthermore, Western blot analysis showed that in comparison with the Ctrl group, the protein level of the autophagic hallmark proteins Beclin1 and LC3 was markedly up-regulated, while that of p62 was down-regulated in the OGD/R group. After inhibition of Cx32, these alterations were further reinforced (Figure 2F, Supplementary Figure 1). These data indicated that after OGD/R, siCx32 facilitated autophagy, which implicated that autophagy may be a mechanism of its protective action. Furthermore, 3-MA (2.5 mmol/L), an autophagic inhibitor, abolished the role of siCx32 in OGD/R, demonstrating autophagy was involved in siCx32 function. However, without OGD/R stimulus, transfecting siCx32 alone didn’t show any effect on cell viability (Figure 2B) or autophagy (Figure 2C–2F). Therefore, we didn’t choose siCx32 alone group in the following experiments.

### Nur77 translocated from nucleus to mitochondria after the inhibition of Cx32 following OGD/R injury

To reveal the mechanism underlying Cx32-induced autophagy after OGD/R injury, we detected the expression of Nur77 in the SH-SY5Y cells. Results showed that the total expression of Nur77 was obviously up-regulated in the OGD/R group, which was further increased in siCx32 cells (Figure 2G, Supplementary Figure 2A). After transfection with Cx32siRNA, the expression of Nur77 in nucleus was decreased, while that in mitochondria was increased. These results indicated that transfection with Cx32siRNA promoted Nur77 translocation from the nucleus to mitochondria (Figure 2H, Supplementary Figure 2B). To confirm that inhibition of Cx32 protected against neuronal damage by regulating Nur77, siNur77 and siCx32 were transfected simultaneously in the same cell. As shown in Figure 2I, cell viability was obviously reduced in after OGD/R injury. This cytotoxic effect was alleviated in siCx32 cells after OGD/R injury. However, after transfection with siCx32 and siNur77 simultaneously, cell viability was reduced again. To further classify that Nur77’s translocation from nucleus to mitochondria plays a part in the action of Cx32, we applied GF109203X, an inhibitor of the PKC family, to treat siCx32 cells after OGD/R. Inhibition of the PKC family has been demonstrated to greatly reduce Nur77’s translocation from nucleus to mitochondria. As shown in Figure 2I, compared to the siCx32 group, GF109203X obviously reduced cell viability in siCx32 cells after OGD/R. These data indicated that inhibition of Cx32 protected against neuronal damage by promoting Nur77 translocation from nucleus to mitochondria.

### Nur77 attenuated mitochondrial dysfunction after the inhibition of Cx32 following OGD/R injury

**Figure 3:**
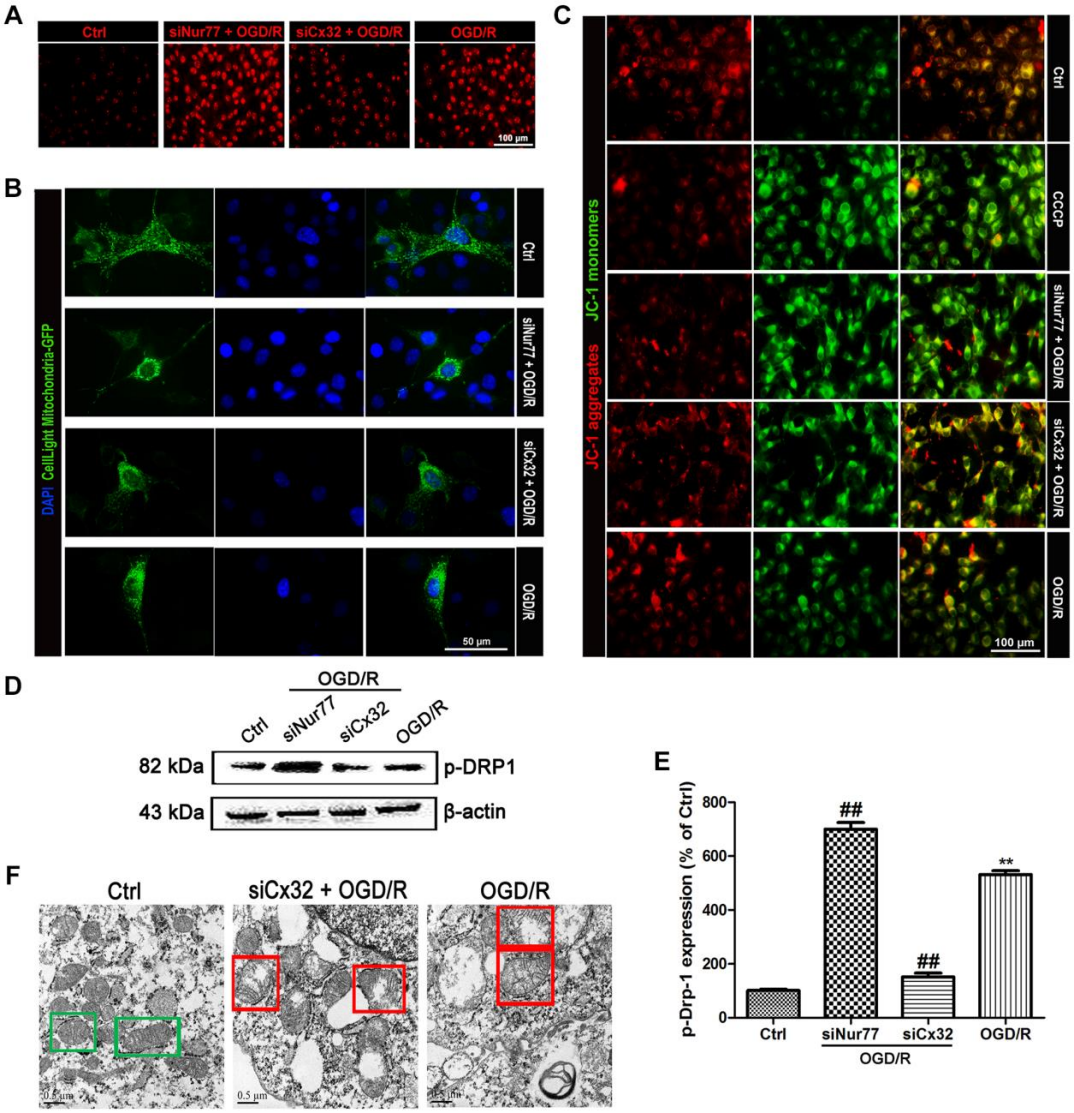
**Nur77 attenuated mitochondrial dysfunction after the inhibition of Cx32 following OGD/R injury.** Cells were transfected with Cx32 siRNA or Nur77 siRNA for 24 h followed by OGD/R (2 h OGD following by 24 h recovery) before harvested. (**A**) The level of ROS was detected by DCF-DA. Scale bars, 100 μm. (**B**) Mitochondria was labeled with GFP (green); cell nuclei were counterstained with DAPI (blue). Photomicrographs were captured under a Nikon Ni-U fluorescence microscope. Scale bars, 50 μm. (**C**) Δψm was detected by JC-1 dye. Images were shown as the ratio of JC-1 aggregates to JC-1 monomers. Scale bars, 50 μm. (**D**–**E**) Representative bands of p-Drp-1, COX4 and TOMM20 protein after OGD/R. Variation in protein loading was determined by blotting with an anti-β-actin antibody. Densitometric scanning of band intensities were calculated as means ± SD (*n* = 3). ^**^p < 0.01 vs. Ctrl group, ^##^*p* < 0.01 vs. OGD/R. (**F**) Images were collected by transmission electron microscope. Green square represents normal mitochondria with cylindrical shape. Their cristae was well-defined, the double membranes was intact and the density was homogenous; Red square represents abnormal mitochondria. Their cristae was disordered, double membrane was discontinuous, electron density was decreased and minor axis was increased consistent with mitochondrial swelling. Scale bars, 0.5 μm.

Multiple studies reported that ischemic stroke injury results in ROS overproduction and cellular component damage, which triggers mitochondrial dysfunction. In the present study, microscopic analysis showed a remarkable elevation of ROS in the SH-SY5Y cells following OGD/R injury. The ROS production was further elevated in siNur77 cell, whereas decreased in siCx32 cells (Figure 3A). Cell Light mitochondria-GFP staining showed that in comparison with the Ctrl group, there was obviously greater perinuclear clustering of mitochondria in OGD/R group. Knock down Nur77 gene further strengthened the perinuclear clustering of mitochondria, whereas it was weakened in siCx32 cells (Figure 3B). In addition, JC-1 was used for detecting the mitochondrial membrane potential (Δψm). In the Control group, JC-1 accumulated in mitochondria as shown by a bright red fluorescence. After OGD/R injury, green fluorescence was increased and red fluorescence was decreased, indicating the rapid depolarization of Δψm. These changes were further strengthened in the Nur77siRNA-transfected cells. In contrast, knock down Cx32 gene obviously prevented OGD/R-induced Δψm dissipation as shown by suppression of green fluorescence and restoration of red fluorescence (Figure 3C). As the mitochondrial markers, the expression of TOMM20 and COX4 were analyzed. As shown in Figure 3D–3E, after OGD/R injury, TOMM20 and COX4 expressions were significantly reduced. Transfection with siCx32 reinforced this reduction, whereas transfection with siCxNur77 obviously reversed this effect. The interaction between mutant proteins and Drp1 probably results in the initiation of abnormal mitochondrial fission. Considering that Ser616 phosphorylation promotes the translocation of Drp-1 to mitochondria. We then detected the level of Phospho-Drp-1 (Ser616) expression. Results demonstrated that knock down Cx32 gene suppressed the increase of p-Drp-1 expression induced by OGD/R injury. In addition, transmission electron microscopy showed abnormal mitochondrial was found in OGD/R cells. Less abnormal mitochondrial were observed in siRNA Cx32 group. These data suggested that the inhibition of Cx32 attenuated mitochondrial dysfunction through the regulation of Niur77, thereby protected against neuronal damage induced by OGD/R (Figure 3F).

### Nur77 activated mitophagy after the inhibition of Cx32 following OGD/R injury

**Figure 4:**
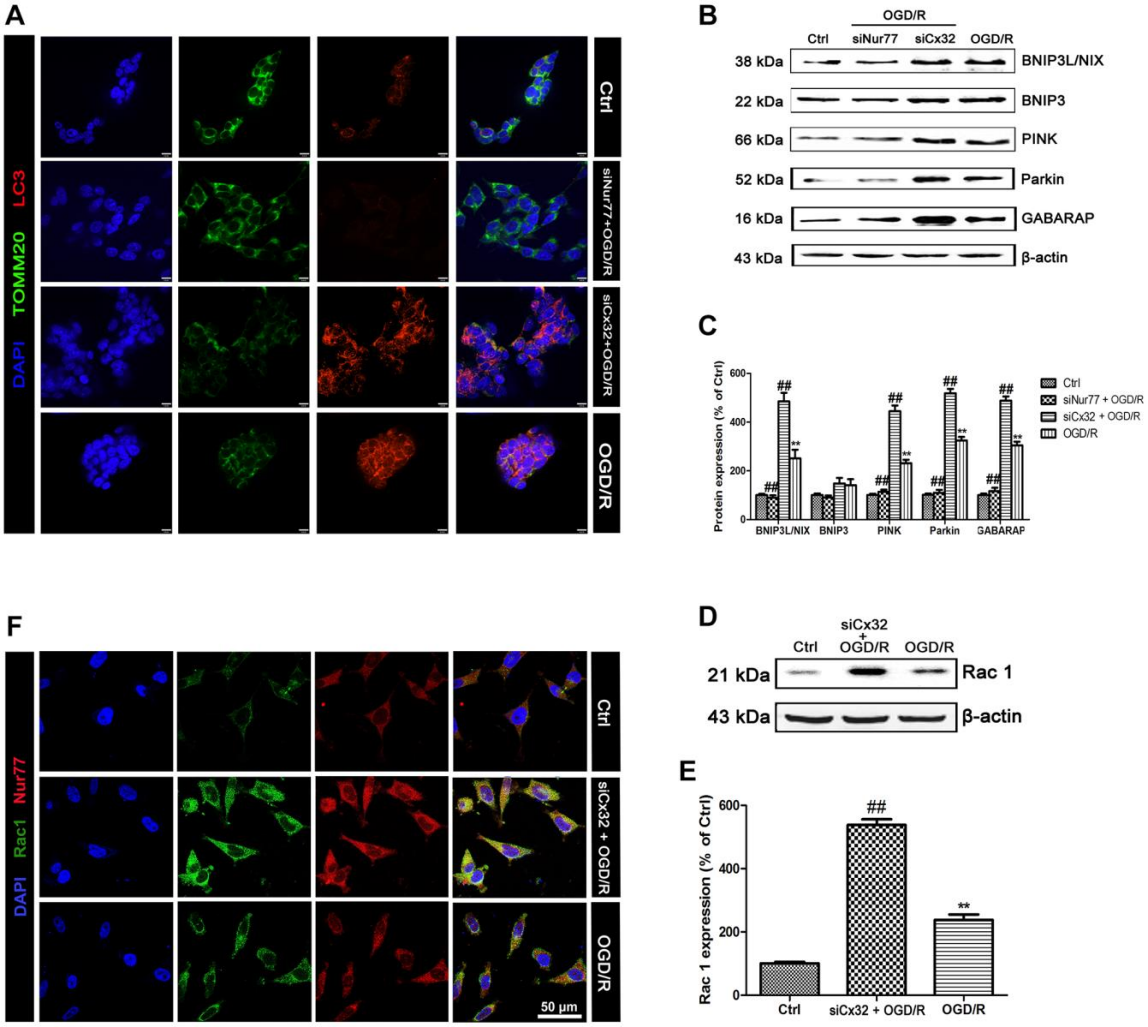
**Nur77 activated mitophagy after the inhibition of Cx32 following OGD/R injury.** Cells were transfected with Cx32 siRNA or Nur77 siRNA for 24 h followed by OGD/R (2 h OGD following by 24 h recovery) before harvested. (**A**) The autophagosome was marked with LC3 (red); mitochondria was marked by TOMM20 (green). Scale bars, 25 μm. (**B**–**C**) Representative bands of BNIP3L/NIX, BNIP3, PINK, Parkin, and GABARAP protein in the cells after OGD/R. (**D**–**E**) Representative bands of Rac1 protein after OGD/R. Variation in protein loading was determined by blotting with an anti-β-actin antibody. Densitometric scanning of band intensities were calculated as means ± SD (*n* = 3). ^**^p < 0.01 vs. Ctrl group, ^##^*p* < 0.01 vs. OGD/R. (**F**) Double labeling of Nur77 and Rac1 using fixed cells of Control, OGD/R or siCx32 group. Scale bars, 50 μm.

To detect mitophagy, LC3 and TOMM20 double staining was applied. As shown in Figure 4A, OGD/R injury increased LC3 and decreased TOMM20 expression in comparison with the Control group, indicating the promotion of mitophagy. Of note, these effects were reversed after transfection with Nur77siRNA, but reinforced after transfected with Cx32siRNA. Furthermore, the mitophagy-related proteins were detected. Results found that OGD/R induced up-regulation of BNIP3L/NIX, PINK, Parkin and GABARAP expression. Knock down Nur77 declined these proteins’ expression, whereas knock down Cx32 up-regulated those. However, there was no difference of BNIP3 expression between groups (Figure 4B–4C).

### Nur77 correlated with Rac1 after the inhibition of Cx32 following OGD/R injury

In this study, Rac1 expression was markedly up-regulated in OGD/R group in comparison with the Control group. After transfection with Cx32siRNA, the level of Rac1 expression was further increased (Figure 4D–4E). To investigate the relationship between Rac1 and Nur77, an immunofluorescent method was used. As shown in Figure 4F, Rac1 immunopositive cells were mainly colocalized with the Nur77 immunopositive cells in the Cx32siRNA-transfected cells, whereas the coimmunoreactive cells were almost diminished in the OGD/R-treated cells. These data indicate that after the inhibition of Cx32, Nur77 translocated from nucleus to mitochondria, where it correlated with Rac1, and further stimulates mitophagy, finally prevented neuronal damage.

### I/R induced up-regulation of Cx32 expression and activation of autophagy

**Figure 5:**
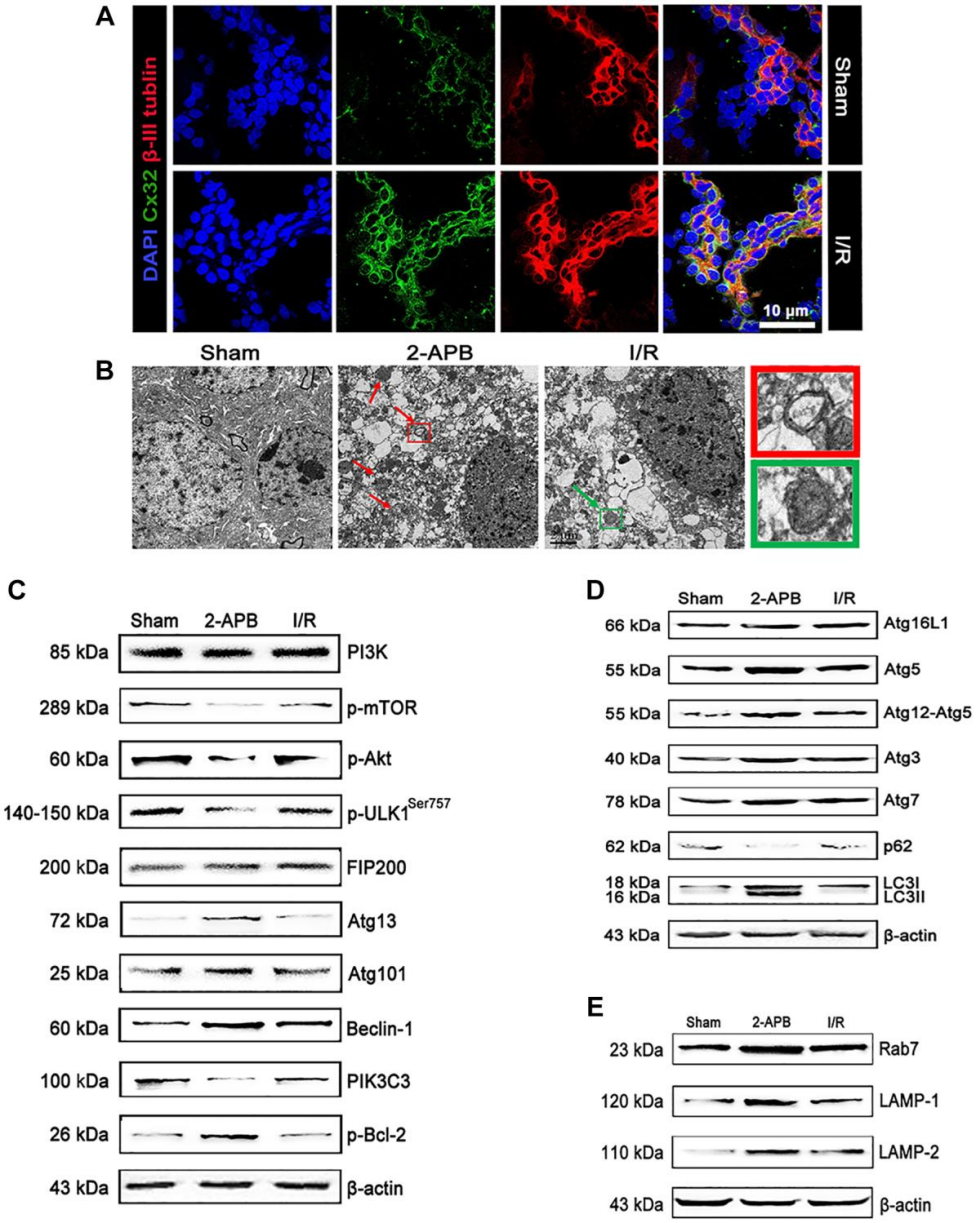
**I/R induced up-regulation of Cx32 expression and activation of autophagy.** (**A**) Double labeling of Cx32 and β-tubulin in the brains of Sham or I/R group. Scale bars, 50 μm. (**B**) Images acquired by transmission electron microscope. The arrow marks autophagic vacuoles. (**C**–**E**) Representative bands of autophagy-related proteins after I/R. Variation in protein loading was determined by blotting with an anti-β-actin antibody.

To verify the results from the *in vitro* experiments, we then investigate the role of Cx32 *in vivo* using a MCAO model. In consistent with *in vitro* results, double immunostaining showed that Cx32 can be expressed in the brain of rats with β-III Tubulin. After I/R injury, the level of Cx32 expression was obviously increased in comparison with Sham group (Figure 5A). To explore the critical role of Cx32 *in vivo* after I/R injury, a Cx32 inhibitor 2-APB was used. As shown in Figure 5B, more autophagic vesicles were formed in the cytoplasm of neurons in I/R group, which was further increased after 2-APB treatment. Considering PI3K/Akt/mTOR pathway, ULK1/Atg13/FIFP200/Atg101 and PIK3C3/Beclin-1/Bcl-2 play vital roles in regulating autophagy, the effect of Cx32 on these pathways was investigated. As shown in Figure 5C, I/R decreased p-mTOR, p-Akt and ULK1 expression. I/R injury activated the PIK3C3/Beclin-1/Bcl-2 pathway as evidenced by an increase of p-Bcl-2 and Beclin-1 protein expression, a decline of PIK3C3 protein expression. In comparison with I/R group, the same changes of these proteins above were reinforced in the 2-APB-treated group. Moreover, we detected the levels of the autophagy markers. Our data showed that LC3-II and Atg5 protein expressions were up-regulated, whereas that of p62 was down-regulated in the I/R group (Figure 5D). These changes were also reinforced in the 2-APB-treated group. As markers of lysosomes, Rab7, LAMP1 and LAMP2 expression was further elevated after 2-APB treatment (Figure 5E). The above results confirmed what we found from the *in vitro* experiment, that is the activation of autophagy after inhibiting Cx32 expression during I/R.

### 2-APB decreased neuronal damage after I/R injury

**Figure 6:**
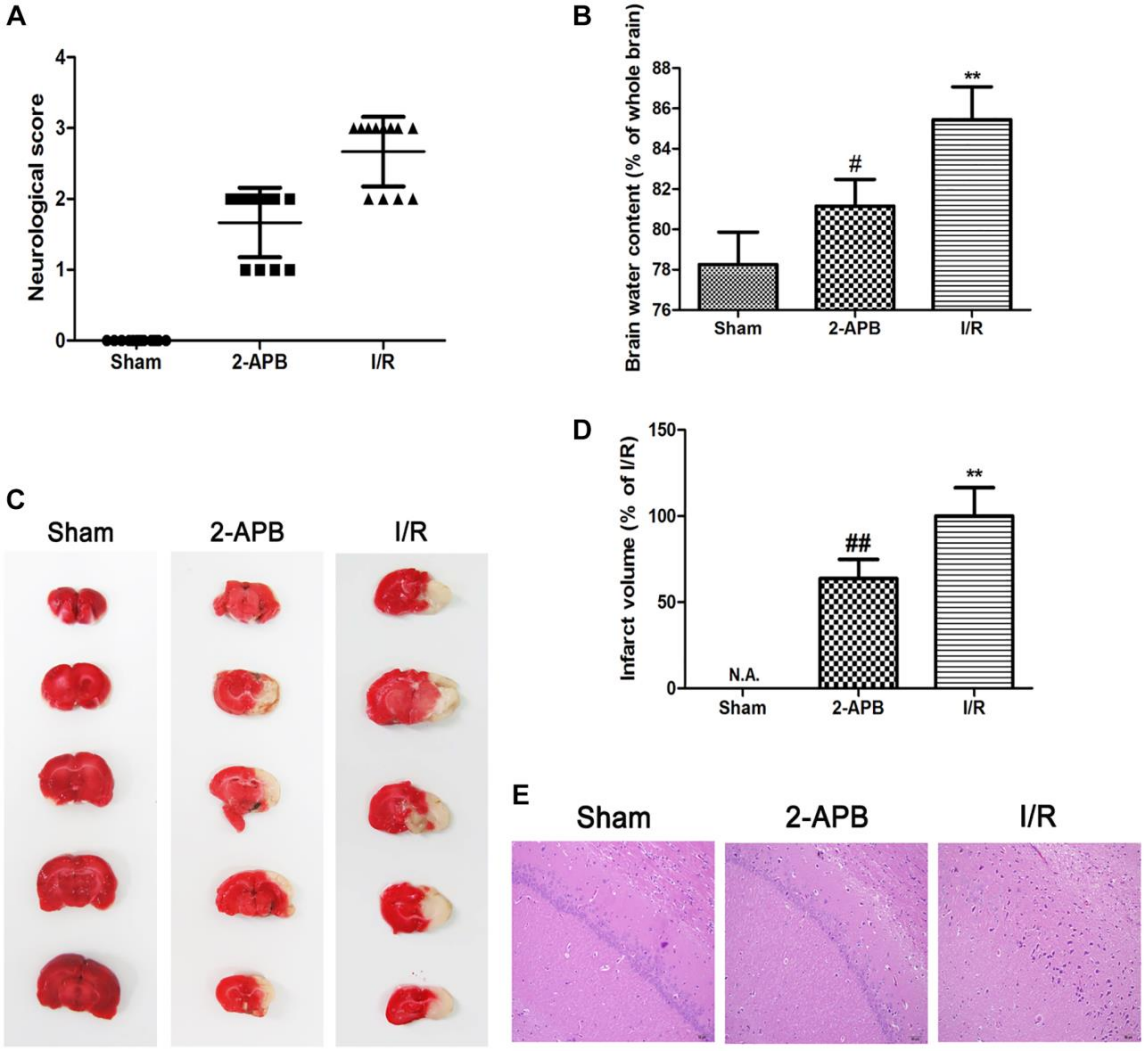
**2-APB decreased neuronal damage after I/R injury.** (**A**) Neurologic scores were evaluated at 72 h after reperfusion (*n* = 12). (**B**) Brain edema was examined at 72 h after reperfusion. Data are expressed as means ± SD (*n* = 6). (**C**–**D**) The infarct area was observed by TTC staining. The infarct size was shown as the ratio of corrected infarct area to whole brain area. Data are expressed as means ± SD (*n* = 6). ^**^p < 0.01 vs. Sham, ^##^*p* < 0.01 vs. I/R. (**E**) The brain sections of rats were stained with H&E staining (magnification, ×200).

Since 2-APB could activate autophagy, we next investigated if it shows neuroprotective effect against I/R injury. Neurological deficit was assessed at 72 h after reperfusion. I/R-operated rats showed prominent neurological deficits in comparison with the Sham group. However, neurological scores were obviously decreased in 2-APB-treated groups (Figure 6A). Consistently, the water content of the ischemic brains was reduced after 2-APB treatment (Figure 6B). TTC staining showed that there is no histological evidence for infarction in the Sham group. I/R injury-induced visible infarction in the ipsilateral hemisphere at 72 h after I/R. 2-APB significantly decreased infarct size in comparison with the Sham group (Figure 6C–6D). Hematoxylin and eosin (H&E) staining showed that there was no neuronal damage or inflammatory cells in the Sham group. The nucleus of the neuron is located in the center of the cell and the staining is clear. After I/R injury, a large number of degenerative and necrotic cells were found in brain tissue, which showed nuclear pyknosis and vacuolation. These results indicated inflammatory infiltration and edema around blood vessels. Interestingly, in the brains of 2-APB-treated rats, the injury degree of neurons was lighter than that of I/R group (Figure 6E).

### 2-APB activated mitophagy through promoting Nur77 translocation from nucleus to mitochondrial after I/R injury

**Figure 7:**
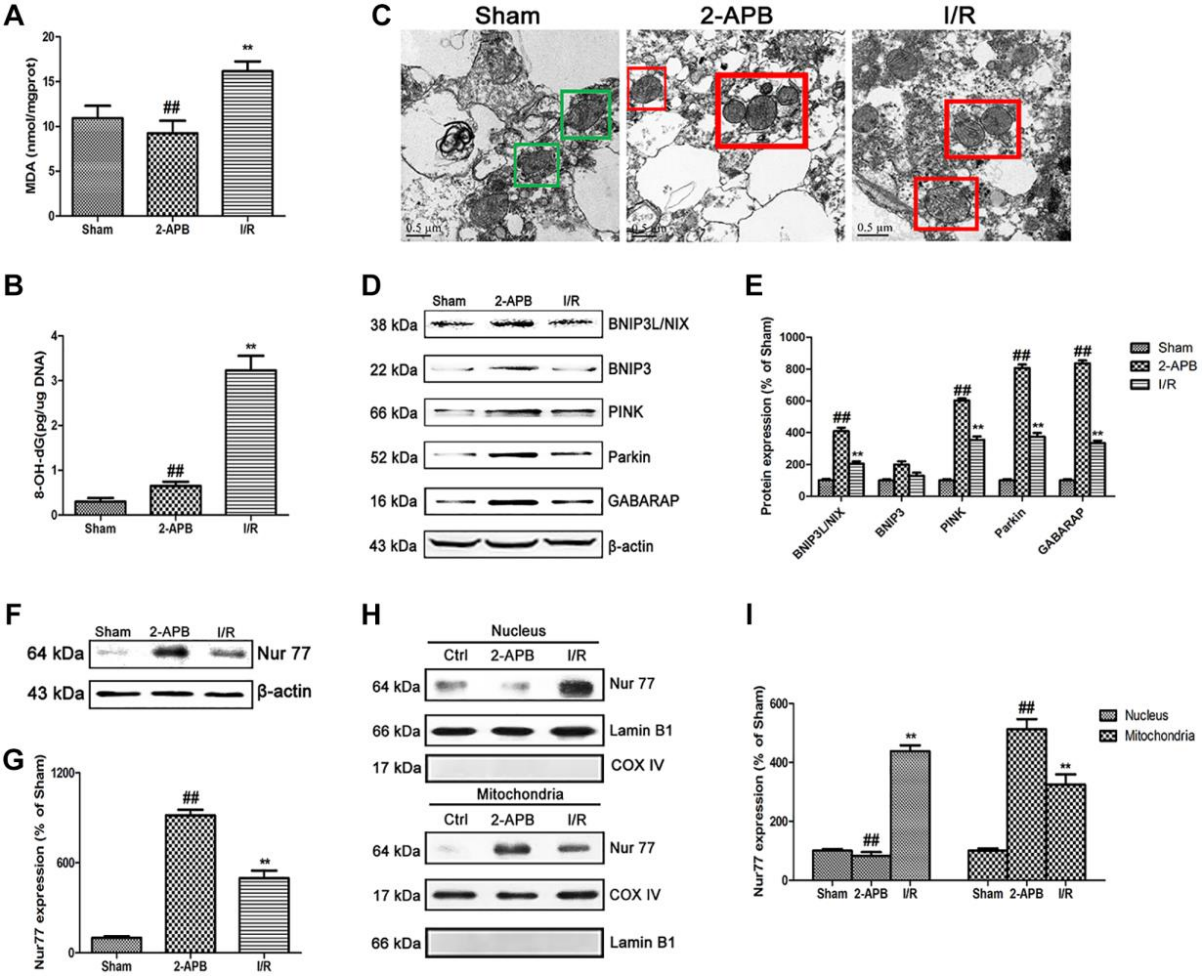
**2-APB activated mitophagy through promoting Nur77 translocation from nucleus to mitochondrial after I/R injury.** (**A**) and (**B**) The levels of MDA and 8-OH-dG were examined at 72 h after reperfusion. (**C**) Images acquired by transmission electron microscope. Green square represents normal mitochondria with cylindrical shape. Their cristae was well-defined, the double membranes was intact and the density was homogenous; Red square represents abnormal mitochondria. Their cristae was disordered, double membrane was discontinuous, electron density was decreased and minor axis was increased consistent with mitochondrial swelling. Scale bars, 0.5 μm. (**D**–**E**) Representative bands of mitophagy-related proteins in brain tissues after I/R. (**F**–**G**) Representative bands of Nur77 protein in brain tissues after I/R. (**H**–**I**) Mitochondrial translocation of Nur77 was examined by Western blot after I/R. Mitochondria Nur77 expression was normalized against COX IV. Nucleus Nur77 expression was normalized against Lamin B1 expression. Variation in protein loading was determined by blotting with an anti-β-actin antibody. Densitometric scanning of band intensities were calculated as means ± SD (*n* = 3). ^**^p < 0.01 vs. Sham group, ^##^*p* < 0.01 vs. I/R.

To explore the mechanism of 2-APB against I/R injury, we firstly measured 2 established oxidative stress markers MDA and 8-OH-dG. In consistence with *in vitro* results, 8-OH-dG and MDA content were obviously increased in the I/R group. However, after 2-APB treatment, these changes were reversed (Figure 7A–7B). Then, transmission electron microscopy was employed to examine mitochondrial. As shown in Figure 7C, I/R damage leads to mitochondrial dysfunction, cristae disorder, double membrane discontinuity, decreased electron density, increased short axis consistent with mitochondrial swelling. In the brains of 2-APB-treated group, more normal mitochondrial was found in comparison of the I/R group. The mitochondrial autophagy marker molecules were then examined by Western blot. Results found that I/R-induced up-regulation of BNIP3L/NIX, PINK, Parkin and GABARAP expression, which was further elevated after 2-APB treatment. However, there was no obviously difference of BNIP3 expression between groups (Figure 7D–7E). Then we examined the expression of Nur77 in the brains of all groups. Results showed that after 2-APB treatment, the expression of Nur77 was further up-regulated (Figure 7F–7G). Besides, 2-APB treatment promoted Nur77 translocation from nucleus to mitochondrial (Figure 7H–7I).

### 2-APB inhibited the dissociation of Nur77 and Rac1 after I/R injury

**Figure 8:**
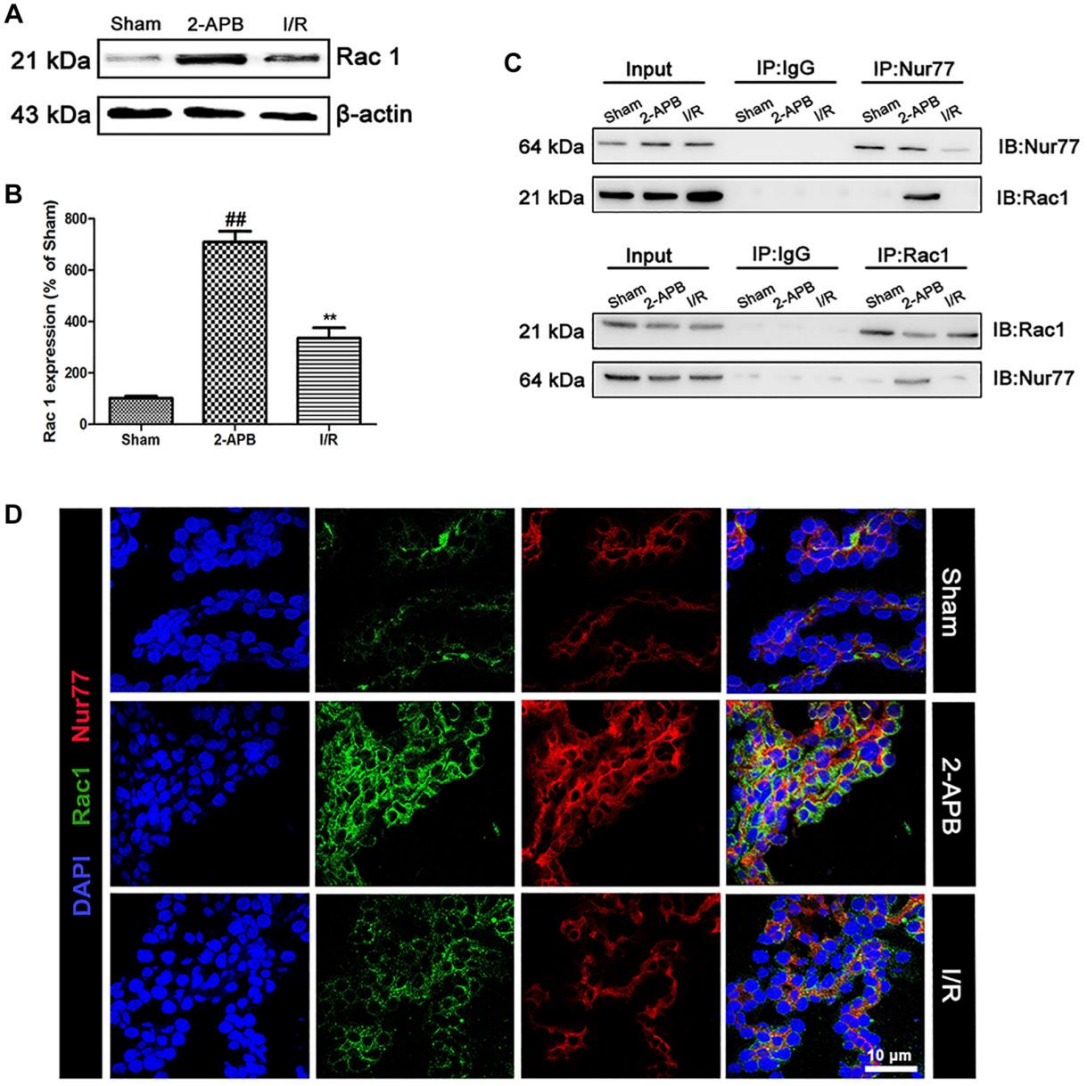
**2-APB prohibited the dissociation of Rac1 and Nur77 after I/R injury.** (**A**–**B**) Representative bands of Rac1 protein in brain tissues after I/R. Statistical results from the densitometric measurements after normalization against β-actin were calculated as means ± SD (*n* = 3). ^**^p < 0.01 vs. Sham group, ^##^*p* < 0.01 vs. I/R. (**C**) Detect the correlation between Nur77 and Rac1 by a Co-IP assay. (**D**) Double labeling of Rac1 and Nur77 in the brain tissues of Sham or I/R group. Scale bars, 50 μm.

To confirm the relationship between Nur77 and Rac1 that we concluded from the *in vitro* data, we firstly examined the expression of Rac1 in the brains of all groups. As shown in Figure 8A–8B, after 2-APB treatment, the level of Rac1 expression was further increased (Figure 8A–8B). Then we detected the possibly physical correlations between Nur77 and Rac1 in the brains of rat. As shown in Figure 8C, there existed no correlation between Nur77 and Rac1 in the I/R group, whereas it was detected after treatment with 2-APB. Further immunofluorescent assay revealed that after 2-APB treatment, Rac1 immunopositive cells were mainly colocalized with the Nur77 immunopositive cells. However, I/R injury declined these coimmunoreactive cells (Figure 8D). These data indicated that after I/R injury, the inhibition of Cx32 promoted Nur77 translocation from the nucleus to the mitochondria, where it correlated with Rac1, consistent with the *in vitro* results.

## DISCUSSION

Connexins are of vital importance in cell differentiation, organ or tissue homeostasis [20–21]. As a member of the connexin family, Cx32 expression was significantly elevated in the ischemic brain tissue of the elderly, indicating a close relationship between Cx32 and ischemic brain injury [22]. However, there is litter literature elucidating the mechanism of Cx32 underlying cerebral ischemia. In this study, we mainly focused on the Cx32-dependent modulation of neuronal autophagy following ischemic stroke *in vitro* and *in vivo*.

Firstly, double immunostaining of Cx32 and β-tubulin revealed the existence of Cx32 in the neuronal cells, which expression was significantly up-regulated after OGD/R injury. It has been reported that although ischemic nuclear necrosis often occurs in neurons after ischemic injury, apoptosis and autophagy are more common in the ischemic penumbra [15, 23]. I/R-induced autophagy protects neuronal damage from neurodegenerative diseases [24], but excessive autophagy leads to neuronal cell death [25]. As we known, LC3-I converts to LC3-II to induce the formation of autophagosome. Therefore, the LC3-II/I ratio is used as a marker for evaluating the autophagy level [26]. In our study, we demonstrated that OGD/R injury induced the activation of autophagy by the elevation of Becilin-1 and LC3 expression. In concurrent with previous studies [27], inhibiting autophagy with 3-MA reinforced the cell injury induced by OGD/R, suggesting a protective effect of autophagy in OGD/R insult. To clarify the correlation between Cx32 and autophagy, cells were transfected with the Cx32-specific siRNA. Results showed that after the inhibition of Cx32, autophagy was further activated as evidenced by the increase of autophagic vacuoles, fluorescent substances AO and MDC, along with the up-regulation of Beclin-1 and LC3 protein expression. All these data above indicated that inhibition of Cx32 expression exerts neuroprotective effect during OGD/R may be attributed to the activation of autophagy.

Nur77 plays a pivotal role in many cellular processes by responding to a variety of stimuli (e.g., cytokines, inheritance, apoptotic signals, etc.) [28–30]. Genetic studies have shown that Nur77 is involved in the inflammatory response, and its prominent role is the protection of arthritis, atherosclerosis, inflammatory bowel disease, obesity, asthma and diabetes [31–36]. However, the function of Nur77 in cerebral ischemia has rarely been investigated. Current data showed that after OGD/R, Nur77 protein expression was obviously up-regulated. To confirm the role of Nur77 in OGD/R injury, cells were transfected with the Nur77-specific siRNA. We observed that knock down Nur77 gene aggravated OGD/R-induced neuronal damage, indicating that Nur77 might be a positive role during OGD/R. Besides, after transfection with siCx32RNA, the expression of Nur77 further up-regulated, and it could translocate from nucleus to mitochondrial. When ischemic stroke-induced oxidative stress occurs, a larger number of ROS are formed, which damage mitochondrial function. Based on the above results, we hypothesized that Nur77’s translocation to mitochondrial might alleviate its dysfunction, which may contribute to the neuroprotection after the inhibition of Cx32. The hallmarks of mitochondrial dysfunction include clustering of mitochondria, decreased membrane potential, production of ROS and so on. During mitochondria dysfunction, damaged mitochondria form a perinuclear cluster, which is subsequently eliminated. Microscopic analysis revealed that knock down Nur77 gene aggravated mitochondrial dysfunction evidenced by more production of ROS, greater perinuclear clustering of mitochondria, and lower mitochondrial membrane potential. However, knock down Cx32 alleviated this damage. Multiple studies reported that increased mitochondrial fission primarily contributes to mitochondrial dysfunction [37]. Drp1 belongs to the dynamin family of large GTPases. After translocation to mitochondrial, Drp1 induces mitochondrial fission and further dysfunction. Finally, the mitochondrial membrane potential was dissipated. Current data demonstrated that knock down Nur77 strengthened the increase of p-Drp1 expression after OGD/R, whereas knock down Cx32 decreased its expression. Furthermore, under transmission electron microscope, more abnormal mitochondrial was found in siNur77 cells, which was decreased in siCx32 cells. All the data above suggested that inhibition of Cx32 promoted Nur77 translocation from nucleus to mitochondrial, attenuated mitochondrial dysfunction, thereby protected against neuronal damage.

Dysfunctional mitochondrial can be cleared by selective autophagy, which termed “mitophagy” [38–39]. BNIP3L and NIX can recruit LC3 and GABARAP to mitochondria, where they promote the formation and maturity of autophagosome [40–41]. After depolarization of a large number of mitochondria, PINK1 phosphorylated and recruited ubiquitin and Parkin on the surface of damaged mitochondria. In the present study, OGD/R activated mitophagy by up-regulating BNIP3L/NIX, PINK, Parkin and GABARAP expression. In siNur77 cells, the expression of these proteins was decreased, whereas in siCx32 cells, they further were increased. These data indicated that after the inhibition of Cx32, Nur77 induced mitophagy to protect against OGD/R injury.

As a binary switch, rac1 can regulate mitochondrial behavior [42]. It plays an important role in multiple signaling pathways involved in cerebral ischemia [43–44]. Knocking out or pharmacological inhibition of Rac1 reduced the degree of brain tissue damage and edema after ischemia [45–46]. Thus, we hypothesized that after Nur77 translocated from the nucleus to the mitochondria, it might correlate with Rac1 and further prevented neuronal damage. To verify this hypothesis, we first detected the expression of Rac1 after OGD/R. Results showed that knock down Cx32 gene strengthened the up-regulation of Rac1 expression after OGD/R injury. In addition, immunostaining analysis showed that OGD/R injury broke the correlation between Nur77 and Rac1, which was restored in siCx32 cells. These data suggested that after the inhibition of Cx32, Nur77 translocated from nucleus to mitochondrial, where it correlated with Rac1, thereby prevented against neuronal damage.

On the basis of the *in vitro* findings, we next carried out further studies *in vivo* using a rat model of focal cerebral I/R. In consistent with the *in vitro* results, we demonstrated the existence of Cx32 in the brains of rat and its relationship with autophagy after I/R injury. To verify the role of Cx32 in neuronal damage *in vivo*, a Cx32 inhibitor 2-APB was used. A research study showed that 2-APB can directly and reversibly inhibit connexin channels composed of Cx26 and/or Cx32 through the carboxyl terminal domain [47]. Kuo Du et al. did a similar study [48]. The meaningful finding of this report is an inhibitor of Cx32-gap junction, which is named 2-aminoethoxy-diphenyl-borate (2-APB). Therefore, we chose 2-APB to study the role and mechanism of Cx32 in ischemic stroke *in vivo*. Current data showed that 2-APB not only decreased the infarct size and brain water content, but also improved the neurological deficit after I/R. In addition, transmission electron microscopy showed that after 2-APB treatment, the autophagic vacuoles were significantly increased. PIK3C3/Beclin-1/Bcl-2 is crucial to the formation of the autophagy complex, which can be initiated by ULK1/Atg13/FIFP200/Atg101. PI3K/Akt/mTOR pathway plays a key role in modulating autophagy. Atg12-Atg5 complex promotes protein-lipid conjugation in autophagosome formation. Atg7 plays a critical role in the autophagosome membrane’s elongation and closure [49–50]. When autophagy is suppressed, p62 accumulates. Meanwhile, when autophagy is activated, p62 levels are decreased [51]. In this study, I/R injury remarkably up-regulated the levels of Beclin-1, LC3-II, and Atg5 in the brain of rats. Meanwhile, I/R injury significantly promoted autophagy via suppressing the PI3K signal cascade. These changes were reinforced in 2-APB-treated group. Rab7 is essential for complete autophagic flux [52]. Our observation found that treatment with 2-APB strengthened I/R-induced up-regulation of Rab7, LAMP1, and LAMP2 expression to promote autolysosome formation. All data above revealed that the neuroprotection of 2-APB might be attributed to the activation of autophagy. In consistent with the *in vitro* findings, after I/R injury, 2-APB promoted Nur77 translocation from nucleus to mitochondrial, where it correlated with Rac1, initiated mitophagy, cleared off dysfunctional mitochondrial, thereby protected against cerebral ischemia.

**Figure 9:**
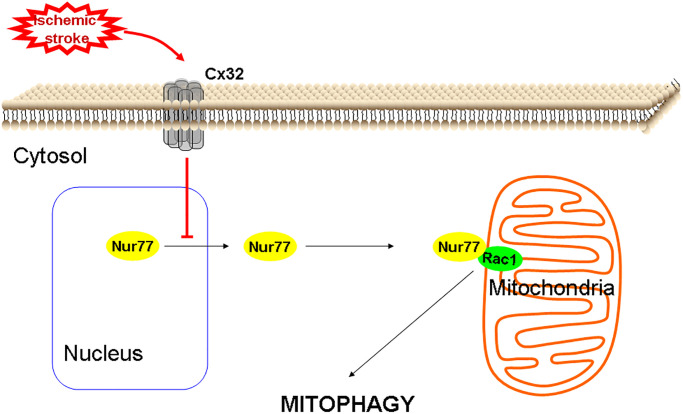
A proposed scheme shows that Cx32 acted as a regulatory factor of Nur77controlling neuronal autophagy in the brains.

In conclusion, the current study evaluated the relationship between Cx32 and autophagy in cerebral ischemia *in vitro* and *in vivo*. Current data suggested that Cx32 acted as a regulatory factor of Nur77controlling neuronal autophagy in the brains. Specifically, inhibition of Cx32 shows neuroprotection through up-regulating Nur77 expression, promoting its translocation from nucleus to mitochondrial, inducing its correlation with Rac1, activating mitophagy to clearance dysfunctional mitochondrial (Figure 9). In different forms of brain injury, the expression level of connexin is different, and this mechanism may be very meaningful in the diseased brain [53]. Despite the above findings, further experiments are needed to reveal the mechanism underlying the effect of Cx32 on autophagy in ischemic stroke. Following experimental plan is to determine how Nur77 may promote autophagic flux; identify regions in Nur77 responsible for binding Rac1 and regions in Rac1 that are required for its correlation with Nur77.

## MATERIALS AND METHODS

### Cell culture and treatment

Human SH-SY5Y cells were purchased from Kaiji Institute of Biotechnology (Nanjing, China). Cells were cultured at 37°C and 5% CO_2_ in DMEM added with penicillin, streptomycin, glutamine and 10% FBS. OGD/R modeling method can be referred to our previous research [54]. In brief, the glucose-free balanced salt solution was bubbled with an anaerobic gas mix (95% N2, 5% CO2). Cells were incubated for 2 h with the solution in an anaerobic chamber (OGD). After replacing the exposure solution with normal DMEM, cells were returned to the normoxic condition for 24 h to terminate OGD injury (recovery, R).

### Cell viability assessment

SH-SY5Y cells were seeded on collagen-coated 96-well plates (10^4^ cells/well). MTT method was applied to measure cell viability at 24 h post-OGD [54].

### Immunofluorescent staining

The SH-SY5Y cells (2 × 10^5^ cells/well) were seeded onto a 3 cm-diameter culture dish and cultured 24 hours. Immunofluorescent staining was conducted in accordance with our previous studies [55]. Primary Abs used were Cx32 (Abcam, ab66613), Nur77, LC3 (Santa Cruz Biotechnology, sc365113, sc271625), Rac1 (Movus, NBP2-67091), β-tubulin (CST4466).

### Transient transfection of siRNA interference

The SH-SY5Y cells were seeded onto a 10 cm dish (2 × 10^6^ cells). The transient transfection of siRNA was performed in accordance with our previous research [54]. In accordance to manufacturer’s instructions, human Nur77, Cx32 and a nonspecific siRNA control (Santa Cruz Biotechnology, Santa Cruz, CA, USA) were used for transfecting the cells with Lipofectamine 2000 transfection reagent (Invitrogen).

### Monodansylcadaverine (MDC) and acridine orange (AO) staining

SH-SY5Y cells were grown in 96-well plates (10^4^ cells/well). In accordance to manufacturer’s instructions, MDC and AO staining were carried out using the kits from Kaiji Institute of Biotechnology (Nanjing, China).

### Western blot analysis

Western blot analysis was conducted in accordance to our previous research [55]. Primary Abs used were Cx32 (ab66613), Nur77 (sc365113), Rac1 (NBP2-67091), p-Bcl-2 (ab73985), p-Drp-1(Ser 616) (CST3455), COX4(CST4850S), α-tubulin (CST 3873), PI3K (CST4257), mTOR (CST5536), p-Akt (CST4060), LC3 (ab3868), p62(CST8025S) , TOMM20(ab56783), ULK1 Complex Ab sampler kit (CST46486), autophagy Ab sampler kit (CST4445), Rab7 (ab137029), LAMP1(ab24170), LAMP2 (ab25631), BNIP3L/NIX (CST12396), BNIP3 (CST44060), PINK (ab23707), Parkin (ab15954), GABARAP (CST13733), and β-actin (CST3700). Proteins were observed with a ECL detection system. A Tanon 5200 Multi (Tanon Science and Technology, Shanghai, China) scanning signal is used for density analysis. Western analysis results reported in this paper are representative at least 3 times. Values are expressed as a percentage of the corresponding Ctrl or Sham value.

### RT-PCR analysis

RT-PCR analysis was performed in accordance with our previous study [56]. The primers are as follows: Cx32: forward, 5′-AGGATGGCTCCCTGAAAGAC-3′ and reverse, 5′-GATGGGAGGTTGCCTGGTAT-3′.

### ROS measurement

SH-SY5Y cells were seeded in a 24-well plate (5 × 10^4^ cells/well) and transfected with Cx32siRNA or Nur77siRNA. ROS generation was measured according to our previous studies [56].

### Subcellular fractionation

The proteins of cytosolic, mitochondria and nuclear fractions were extracted by multiple centrifugation. The mitochondria/nuclei isolation kit was purchased from Kaiji Institute of Biotechnology (Nanjing, China). All subsequent steps were carried out with cold solutions and centrifugation was performed at 4°C.

### Quantification of autophagy

Quantification of autophagy was conducted in according to previous study [56]. SH-SY5Y cells were plated at the desired density with sufficient time to adhere. To ensure a homogenous solution, LC3-green fluorescent protein (GFP) reagent and mitochondria-red fluorescent protein was mixed many times by inversion. Then, they were added to the cells with or without Cx32siRNA. The cells were put back to the incubator for 1 d. Add 50 nM LysoTracker Deep Red was into the cells for incubating 1 h.

### Evaluation of Δψm

SH-SY5Ycells were seeded on coverslips in 24-well plates (5 × 10^4^ cells/well). Cells were transfected with or without Cx32siRNA for 24 h. Then, cells were measured by JC-1 staining according to previous study [56].

### Transmission electron microscopy

SH-SY5Y cells were grown in 60mm dishes (8 × 10^5^ cells). After centrifugation, SH-SY5Y cells were obtained, fixed in PBS (pH 7.3) supplemented with 2.5% glutaraldehyde and then 1% OsO_4_ supplemented with 0.1% potassium ferricyanide for 30 min. By an ascending series of ethanol (30%–90%) and dry acetone, the cells were dehydrated and embedded in epoxy resin. Brain slices were fixed in 4% glutaraldehyde for 1 h and treated post-fixation with 1% OsO_4_ for 2 h, before dehydration in an ascending graded ethanol series and embedding in epoxy resin. After staining the ultrathin sections (70 nm) with uranyl acetate and lead citrate, the results were observed using JEM2000EX transmission electron microscope (JEOL, Tokyo, Japan).

### Animals

All SD rats were male, weighing 250 ~ 300g (B&K Universal Group Limited, Shanghai, China). During the experiment, the animals were treated strictly in accordance with the National Institutes of Health Guidelines for the Care and Use of Laboratory Animals. All procedures were carried out under the supervision of Animal Protection and Utilization Committee of China Pharmaceutical University.

### MCAO and drug administration

Rats were anesthetized with isoflurane followed by MCAO surgery as described before [54]. The control group was treated with sham operation. All rats were randomly divided into three groups (*n* = 12): (1) Sham group; (2) Model group (I/R); (3) I/R + 2-APB (4 mg/kg, i.p.) group (2-APB). At the beginning of reperfusion, rats were given 2-APB every day for 3 consecutive days.

### Evaluation of neurological deficit

The degree of neurological deficit was observed at 72 h after reperfusion by a blinded observer in accordance to Longa’s method [57].

### Evaluation of infarct size and brain-water content

Referring to our previous study [58], the brain tissues were removed quickly. Part of the brain tissues were cut into six coronal sections (2 mm) for TTC staining. Part of the brain tissues were weighed for the water content.

### Histopathological examination

Referring to our previous study [54], the brain tissues were quickly removed and frozen. Part of the brain tissues were sectioned into 20 mm for H&E staining. Part of the brain tissues were kept at −70°C for the detection of MDA and 8-OH-dG levels.

### Coimmunoprecipitation (Co-IP) assay

Co-IP analysis was performed in accordance to our previous study [59]. Briefly, brain tissue lysates were incubated with Rac1 or Nur77 antibody overnight followed by coupling to the immobilized protein A/G agarose beads at 4°C for 4 h. The coimmunoprecipitated Nur77 or Rac1 was finally examined by Western blot with Rac1 or Nur77 antibodies.

### Statistical analysis

All data are expressed as mean ± SD. Statistical analyses were performed with one-way ANOVA followed by Turkey’s post hoc test for multiple comparison tests. Significant differences were accepted when *p* < 0.05.

## Supplementary Materials

Supplementary Figures
