# Supplementary material for: Cx32 inhibits the autophagic effect of Nur77 in SH-SY5Y cells and rat brain with ischemic stroke
Source: Aging (Albany NY). 2021 Sep 22;13(18):22188–207. doi: 10.18632/aging.203526 (PMC8507301; doi:10.18632/aging.203526)
Supplement: Supplementary Figures [file aging-13-203526-s001.pdf]

SUPPLEMENTARY FIGURES

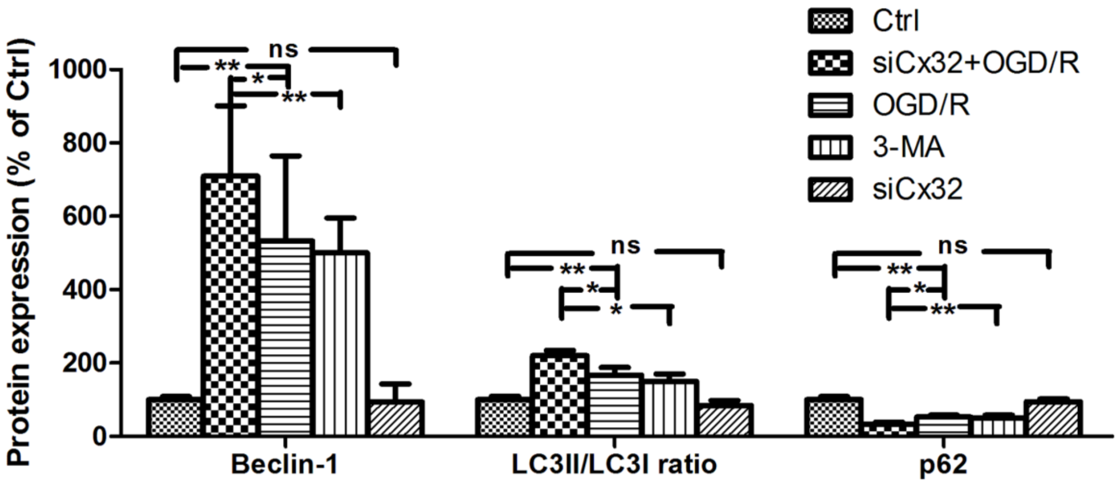

Supplementary Figure 1. Inhibition of Cx32 activated autophagy after OGD/R injury. Representative bands of Beclin1, LC3 and p62 protein in siCx32 cells after OGD/R. Variation in protein loading was determined by blotting with an anti-β-actin antibody. Densitometric scanning of band intensities were calculated as means ± SD (n = 3). $^{**}p < 0.01$, $^{*}p < 0.05$.

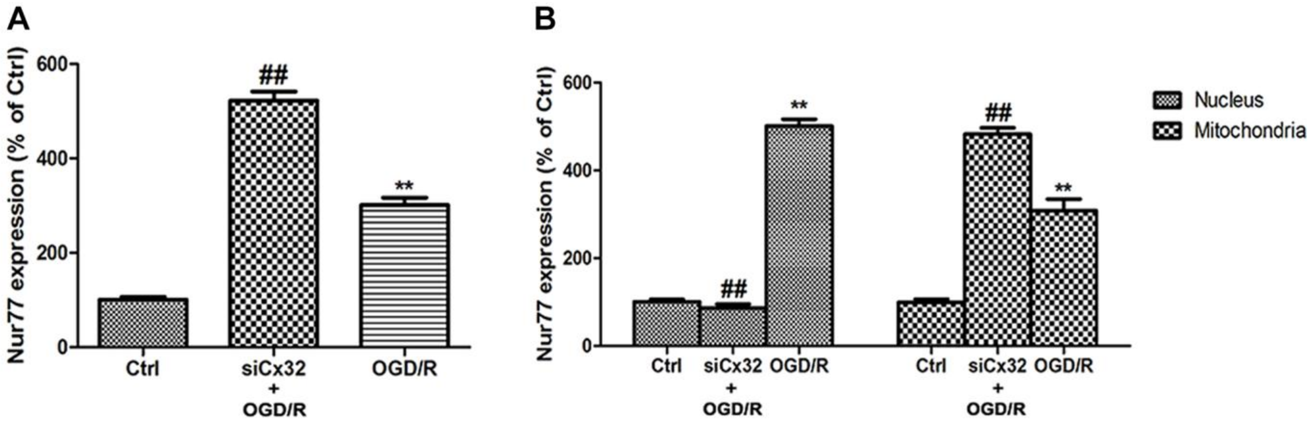

Supplementary Figure 2. Nur77 translocated from nucleus to mitochondria after the inhibition of Cx32 following OGD/R injury. (**A**) Representative bands of Nur77 protein in the cells after OGD/R. (**B**) Mitochondrial translocation of Nur77 was detected by Western blot after OGD/R. Mitochondria Nur77 expression was normalized against COX IV. Nucleus Nur77 expression was normalized against α-tubulin expression. Variation in protein loading was determined by blotting with an anti-β-actin antibody. Densitometric scanning of band intensities were calculated as means ± SD (n = 3). $^{**}p < 0.01$ vs. Ctrl group, $^{\#\#}p < 0.01$ vs. OGD/R.
